# Root nodule organogenesis: a unique lateral organogenesis in legumes

**DOI:** 10.1270/jsbbs.22067

**Published:** 2023-01-06

**Authors:** Takuya Suzaki

**Affiliations:** 1 Faculty of Life and Environmental Sciences, University of Tsukuba, Tsukuba, Ibaraki 305-8572, Japan; 2 Tsukuba Plant-Innovation Research Center, University of Tsukuba, Tsukuba, Ibaraki 305-8572, Japan

**Keywords:** neofunctionalization, NODULE INCEPTION (NIN), root nodule symbiosis, SCARECROW (SCR), SHORTROOT (SHR)

## Abstract

During the course of plant evolution, leguminous and a few plants species have established root nodule symbiosis (RNS), one of the nitrogen nutrient acquisition strategies based on mutual interaction between plants and nitrogen-fixing bacteria. In addition to its useful agronomic trait, RNS comprises a unique form of plant lateral organogenesis; dedifferentiation and activation of cortical cells in the root are induced upon bacterial infection during nodule development. In the past few years, the elucidations of the significance of NODULE INCEPTION transcription factor as a potentially key innovative factor of RNS, the details of its function, and the successive discoveries of its target genes have advanced our understanding underlying molecular mechanisms of nodule organogenesis. In addition, a recent elucidation of the role of legume SHORTROOT-SCARECROW module has provided the insights into the unique properties of legume cortical cells. Here, I summarize such latest findings on the neofunctionalized key players of nodule organogenesis, which may provide clue to understand an evolutionary basis of RNS.

## Introduction

Nitrogen is an essential macro nutrient for the growth and survival of plants. Despite generally low amounts of nitrogen nutrient in the soil, land plants manage to acquire it by developing various strategies (Oldroyd and Leyser 2020). Among the strategies, root nodule symbiosis (RNS) may be the most specialized way to obtain nitrogen nutrient. Legumes and a few plant species are capable of forming root nodules on their roots, in which plants have a mutual endosymbiotic relationship with nitrogen-fixing bacteria collectively called rhizobia (Oldroyd *et al.* 2011, Roy *et al.* 2020, Suzaki *et al.* 2015). Within nodules, rhizobia provide a nitrogen-fixing product, ammonia, to plants and plants instead provide a carbon source for the rhizobia. RNS enables plants to adapt to nitrogen deficient environments, where non-nodulating plants have difficulty in surviving. During nodule development, plants respond to Nod factors, lipo-chitooligosaccharides, produced by the compatible rhizobia; perception of these factors by receptor kinases induces a signaling cascade in the epidermis of the root. Subsequently, dedifferentiation of some of the cortical cells is induced; these cells with altered cell fate then divide to form the nodule primordia (Suzaki and Kawaguchi 2014, Suzaki *et al.* 2014). Meanwhile, rhizobia invade the dividing cortical cells via a tubular structure called the infection thread (Murray 2011, Suzaki *et al.* 2019). After the colonization within the nodule cells, rhizobia are provided with micro-aerobic conditions suitable for the function of nitrogenase, an enzyme complex responsible for nitrogen fixation. Given the striking feature of RNS in plant nutrient acquisition, understanding its molecular basis may ideally lead to the development of technologies to impart this useful agronomic trait to various crops beyond leguminous species. To this end, it is important to reveal the mechanisms determining how RNS had been born during plant evolution. A plausible hypothesis is that RNS had established by co-opting a set of genes involved in other symbiotic system between plants and arbuscular mycorrhhizal (AM) fungi; the AM symbiosis is an older form of plant-microbe symbiosis than RNS (Kistner and Parniske 2002, Oldroyd 2013). Indeed, there are so called *Common Symbiosis Pathway* (*CSP*) genes; the *CSP* genes basically act in a signaling pathway between symbiont infection and symbiotic organ development by generating calcium signaling and making a read-out, which occurs commonly during the two forms of plant-microbe symbiosis. In the case of RNS, nodule organogenesis is initiated downstream of the calcium signaling. The theory of co-opting genes from AM symbiosis is likely to explain part of the evolutionary basis of RNS; however, to reveal the whole aspect, it is especially important to uncover how RNS-specific processes, including nodule organogenesis and symbiotic nitrogen fixation, had been achieved. Here, I summarize the latest findings, mainly focusing on the functions and regulatory mechanisms of key regulators in nodule organogenesis, and their common and unique functions with non-nodulating plants.

## NODULE INCEPTION

A transcription factor (TF) NODULE INCEPTION (NIN) might be one of the key innovative factors that had contributed to provide ancestral plants with the ability to form nodules. The nodulating and non-nodulating properties of closely-relative species in a nitrogen-fixing clade are correlated to the presence and absence of NIN (Griesmann *et al.* 2018, van Velzen *et al.* 2018). NIN belongs to the NIN-LIKE PROTEIN (NLP) TF family whose members are characterized by the presence of N-terminal conserved domain responsible for the nitrate response, an RWP-RK DNA-binding (RWP-RK) and a PB1 domain involved in protein-protein interaction (Schauser *et al.* 2005). Although NLPs have a pivotal role in mediating nitrate response, nitrate responsiveness is lost in NIN; this may be relevant to relatively lower conservation in some region of the N-terminal domain between NIN and NLPs (Konishi and Yanagisawa 2013, Liu *et al.* 2017, Suzuki *et al.* 2013). In model legumes *Lotus japonicus* and *Medicago truncatula*, loss-of-function mutations in *NIN* abolishes nodulation, whereas constitutive expression of *NIN* induces spontaneous cortical cell division resulting in the formation of nodule-like structures in the absence of rhizobia. Thus, NIN has a necessary and sufficient function for positively regulating nodule organogenesis (Marsh *et al.* 2007, Schauser *et al.* 1999, Soyano *et al.* 2013). Transcriptome analysis shows that nearly 90% rhizobia-inducible gene expression is dependent on NIN in *L. japonicus* (Nishida *et al.* 2021). Indeed, expression of many nodulation-related genes required for nodulation is directly targeted by NIN (Jiang *et al.* 2021, Kawaharada *et al.* 2017, Li *et al.* 2019, Soyano *et al.* 2013, 2014, Xie *et al.* 2012). Interestingly, *LATERAL ORGAN BOUNDARIES DOMAIN 16* (*LBD16*), encoding a TF whose orthologues in non-nodulating plants play a role in lateral root development, is identified as a NIN direct target gene to regulate nodule organogenesis (Goh *et al.* 2012, Schiessl *et al.* 2019, Soyano *et al.* 2019). The finding suggests that lateral root developmental program is recruited by NIN to regulate nodule organogenesis. In legumes, LBD16 has dual functions of regulating RNS and lateral root development, whereas the role of NIN is specific to RNS.

**Fig. 1.**
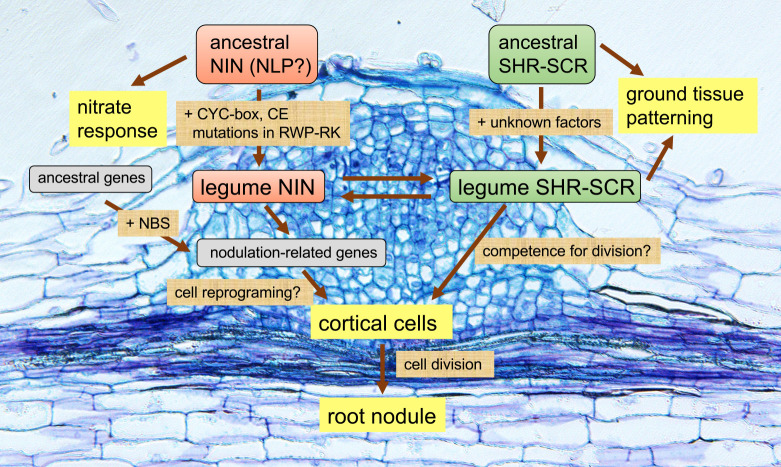
Model of evolutionary basis of root nodule organogenesis. Using legumes as an example, two cis-elements, CYC-box and CE, emerged on the promoter regions of a *NIN* ancestral gene that was probably similar to *NLP*, allowing it to be regulated by pre-existing TFs, such as CYCLOPS. NBS appeared on the promoter regions of some genes, although it is unknown if the event was accompanied by the birth of NIN; the resultant genes with NBS obtained direct molecular link to NIN. The nodulation-related NIN-target genes, including *LBD16*, act as positive regulators of cortical cell division. In addition, unknown mutations provided the legume SHR-SCR module with a neofunctionalized role of regulating nodule organogenesis. The SHR-SCR module is hypothesized to be involved in the regulation of competence for cortical cell division. It is possible that NIN and SHR-SCR module are in a positive regulatory relationship with each other. The background image shows a toluidine blue-stained section of *L. japonicus* root nodule primordium.

Given the role of NLPs as key TFs for nitrate response in many plant species irrelevant to nodulation capability, it is reasonable to conclude that the basal function of NLPs in plants is related to nitrate response (Alfatih *et al.* 2020, Konishi and Yanagisawa 2013, Nishida *et al.* 2021). Thus, the emergence of NIN provides an example of neofunctionalization during plant evolution (Force *et al.* 1999), where after gene duplication one of the NLPs had accumulated mutations and acquired a new function. Then, what mutations would have made NIN such special for it to be able to function in RNS? Clarification of the following two points may lead to address the mystery: 1) NIN had needed to be part of the signaling in RNS, and 2) Genes required for nodulation had needed to be regulated by NIN (Fig. 1).

Regarding the first point, a cis-element named CYC-box is identified on *NIN* promoter region, to which CYCLOPS binds (https://doi.org/10.1101/2022.03.28.486070, Singh *et al.* 2014). *CYCLOPS* is a typical *CSP* gene and encodes TF with coiled-coil motif (Yano *et al.* 2008); the finding indicates the direct molecular link between NIN and a *CSP* gene. Following the calcium signaling induced by rhizobial infection, CALCIUM CALMODULIN-DEPENDENT PROTEIN KINASE phosphorylates CYCLOPS and the phosphorylated CYCLOPS is able to induce *NIN* expression (Singh *et al.* 2014). In addition to its predominant role for nodule organogenesis, NIN has another important role in RNS; it negatively regulates rhizobial infection at epidermis (Liu *et al.* 2019, Yoro *et al.* 2014). The *NIN* expression by CYCLOPS seems to be related to NIN’s role at epidermis rather than that at cortical cells (https://doi.org/10.1101/2022.03.28.486070, Singh *et al.* 2014). For the NIN’s role at cortical cells that is associated with nodule organogenesis, other cis-element, CE (for cytokinin response elements-containing), is recently identified on considerably distantly upstream *NIN* promoter region (Liu *et al.* 2019); in the case of *M. truncatula*, CE is located at 18 kb upstream of the *NIN* coding region, although relevant TF is unknown. It seems that CYC-box and CE are not present on the promoter region of *NLP* genes. Thus, it is likely that the emergence of at least the two cis-elements on the promoter region of ancestral *NIN* might had contributed to place it as part of the signaling in RNS. Now, additional cis-element required for NIN’s proper expression has been identified, suggesting that the regulatory mechanism of *NIN* expression is more complex and under the control of multiple TFs (Akamatsu *et al.* 2021).

The second point can be related to why NIN not NLP can regulate nodulation-related genes expression. For transcriptional regulation, NIN is likely to act as a homodimer, and positively regulates its target genes expression through binding to a cis-element, NBS (for NIN-binding nucleotide sequence); NBS is structurally similar to nitrate-responsive cis-element (NRE), to which NLPs bind (Konishi and Yanagisawa 2010, Nishida *et al.* 2021, Soyano *et al.* 2013). NBS can be divided into two types; the one that is identical to NRE and bound by both NIN and NLP, and the other that is bound specifically by NIN (Misawa *et al.* 2022, Nishida *et al.* 2021, Soyano *et al.* 2015). In *L. japonicus*, the former type has semi-palindromic structure; in contrast, the latter is less palindromic (Nishida *et al.* 2021), indicating that NBS can be more variable patterns than NRE. Notably, the NIN-specific binding site is mainly located on the promoter regions of genes with rhizobia-inducible expression, which may consequently explain why NIN not NLPs can regulate nodulation-related genes expression (Nishida *et al.* 2021). To date, the mechanism by which NIN has a different DNA-binding specificity from NLPs remains elusive, but the clue to solving this problem may lie in clarifying the structural differences in the RWP-RK domain. It is possible that some critical mutations in RWP-RK domain in ancestral NIN had allowed its broader ranged DNA-binding capacity than NLPs. Another mystery is how the NIN-specific binding site came to be on the promoter of nodulation-related genes; especially did this event occur dependently or independently on the birth of NIN? Clarification of these points would greatly advance our understanding of the evolutionary basis of RNS involving NIN.

Determining the function of NLPs and comparing it to NIN would also be useful in understanding the characteristics of NIN. In legumes, NLPs have been shown not only to function as a master regulator of nitrate-induced gene expression as in other plants, but also to be involved in the inhibition of RNS in response to nitrate (Lin *et al.* 2018, Nishida and Suzaki 2018a, 2018b, Nishida *et al.* 2018, 2021). In *L. japonicus*, NLP4 is translocated to the nucleus in the presence of nitrate and NLP4-NIN heterodimer is preferentially formed over NIN homodimer. The NLP4-NIN heterodimer has no or much weaker ability to induce nodulation-related gene expression than NIN homodimer, thus repressing nodulation-related gene expression (Nishida *et al.* 2018, 2021). As mentioned above, some of the NBS are identical to NRE, and NIN can bind to NBS/NRE on the promoter region of nitrate-responsive genes, including *NITRATE TRANSPORTER 2.1* and *NITRITE REDUCTASE* (Misawa *et al.* 2022, Soyano *et al.* 2015). This TF-DNA interaction is not linked to gene expression; rather, it is associated with inhibition of the NLP-mediated induction of nitrate-responsive gene expression.

## SHORTROOT and SCARECROW

Despite the prevailing view that cortex is a differentiated tissue, given that cortex is the site where de novo organogenesis of nodulation takes place, it is suggested that cortical cells of nodulating plants have different anatomical characteristics from those of non-nodulating plants (Geurts *et al.* 2016, Gualtieri and Bisseling 2000). Legume cortical cells may have a propensity to change their fate in response to external stimuli. Although it has long been unknown about the characteristics and regulatory mechanisms of cortical cell formation in nodulating plants, a noteworthy finding has recently been reported. In Arabidopsis, *SHORTROOT* (*SHR*) is expressed in the stele and encoded SHR TF moves to endodermis where it activates *SCARECROW* (*SCR*) expression. SCR has a role to keep SHR protein movement in the endodermis; the SHR-SCR module acts to determine proper ground tissue patterning that consists of one layer of cortex and endodermis (Cui *et al.* 2007, Nakajima *et al.* 2001). In *M. truncatula*, which has 4–5 layers of the cortex, has two AtSHR homologues (MtSHR1 and MtSHR2) (Dong *et al.* 2021). MtSHR1/2 proteins are localized throughout the cortex layers, whereas the *MtSHR1/2* genes expression is restricted to the stele. The extensive MtSHR1/2 protein movements seems to be due to unique amino acid residues in a specific region of MtSHR1/2 that are absent from AtSHR. In addition, with the emergence of novel regulatory cis-elements on the promoter regions of *MtSCR*, *MtSCR* is expressed throughout the cortex layers, which shows a high contrast to *AtSCR* endodermis-restricted expression (Dong *et al.* 2021). These findings indicate that, in *M. truncatula*, the area in which SHR-SCR module functions is spread throughout the entire cortex layers. Interestingly, MtSHR-SCR module is activated by rhizobial infection (Dong *et al.* 2021). Although it remains largely elusive how the activation of MtSHR-SCR module occurs in the context of known nodulation signaling pathway, loss-of-function mutations in *MtSHR1/2* or *MtSCR* impair nodule organogenesis. Moreover, constitutive expression of *MtSHR1* induces spontaneous cortical cell division leading to the formation of nodule-like structures. Thus, these data not only position the SHR-SCR module as a novel regulator of nodule formation, but it also provide a new example of neofunctionalization of genes that might had contributed to the birth of RNS (Fig. 1). Unlike the case of NLP and NIN, in which NIN has acquired a new function while losing its basal function, MtSHR-SCR module maintains its original role of regulating ground tissue patterning.

Based on the currently available data, it is unlikely that the expanded function of the MtSHR-SCR module in entire cortex layers is a determinant of its role of positive regulation in nodulation. Some non-nodulating plants such as rice (*Oryza sativa*) have multiple layers of cortex, which seems to be relevant to the degree of SHR mobile ability (Wu *et al.* 2014). Thus, the number of cortical cell layers does not appear to be directly related to nodulation capacity. Nevertheless, an intriguing hypothesis is that the function of legume SHR-SCR module may be related to the cell division competence for nodule organogenesis (Dong *et al.* 2021). The presence of the SHR-SCR module in the cortex may facilitate changes in the properties of the cortical cells in response to rhizobial infection. Further clarification of the details will help to dissect the uniqueness of the legume cortical cells.

## Conclusions and perspectives

In understanding the evolutionary basis of RNS, the theory that RNS arose on the basis of AM symbiosis has long been a leading hypothesis; however, it alone cannot explain the whole aspects of RNS, including RNS-specific process of nodule organogenesis. Since nodule organogenesis occurs synchronously with rhizobial infection process, it has been often difficult to focus on nodule organogenesis. Nevertheless, in the past few years, the elucidations of the significance of NIN as a potentially key innovative factor of RNS, the details of its function, and the successive discoveries of its target genes have advanced our understanding of the molecular basis of nodule organogenesis, with NIN as a central player. In addition, research has finally begun to tackle the uniqueness of legume cortical cells at the molecular level. However, the substance of dedifferentiation and activation of cortical cells during nodule organogenesis is still unclear; in particular, the cellular and molecular biological entities of reprogramming that alters cell fate remain enigmatic. The recent remarkably progressing single-cell analyses should be powerful approaches for the research of nodule organogenesis. In this regard, initial cell reprogramming event is thought to locally occur in a very small number of cells; hence, elaborated spatiotemporal information must be carefully considered.

Soybean (*Glycine max*) is one of the most important crops for seed protein and oil content. Due to its palaeopolyploid nature and a long-standing difficulty of creating transgenic plants, genetic approaches to reveal molecular mechanisms of RNS have lagged behind that of two model legumes. Now, however, the establishment of transformation technology combined with genome editing has made reverse genetics of soybean possible. Indeed, there are emerging examples of important discoveries on RNS that were first made using soybean (Ji *et al.* 2022, Wang *et al.* 2021). Thus, in the future soybean researches on the molecular mechanisms of RNS will be greatly accelerated and the obtained knowledge possibly directly contributes to agricultural applications.

## Author Contribution Statement

T.S. wrote the manuscript.
